# HIV index testing to improve HIV positivity rate and linkage to care and treatment of sexual partners, adolescents and children of PLHIV in Lesotho

**DOI:** 10.1371/journal.pone.0212762

**Published:** 2019-03-27

**Authors:** Makhahliso Jubilee, Faith Jiyeong Park, Knowledge Chipango, Kenoakae Pule, Albert Machinda, Noah Taruberekera

**Affiliations:** 1 Population Services International, Maseru, Lesotho; 2 Population Services International, Johannesburg, South Africa; Medical University of Warsaw, POLAND

## Abstract

Despite years of HIV testing and other interventions, Lesotho continues to experience an incredibly high HIV burden. Prevalence of HIV among children ages 0–14 years is at 2.1% and 25.6% among adults ages 15–59 years. Among adults living with HIV, 77.2% know their status, 90.2% of those with known HIV positive status are currently receiving ART and 88.3% are virally suppressed. In order to identify adults, adolescents and children at high risk of HIV infection, Population Services International (PSI)/Lesotho with support from the Centers for Disease Control and Prevention (CDC) introduced the HIV Index testing model in 2015. PLHIV recruited for index testing, were accessed through health facilities and community testing at PSI New Start channels in five districts. Consenting index clients received home visits for HIV testing of their biological children and sexual partners with unknown status. Routine monitoring of data gathered between May 2015 and November 2017 was analyzed to assess feasibility of this approach. For HIV index testing, 49.2% of children below 15 years and 37.3% of adolescents ages 15–19 were first time testers while 18.8% of all adults aged 20 years and above tested were testing for the first time. Higher HIV positivity rates among clients tested through the HIV index testing model across all age groups in comparison to other HIV testing models were statistically significant. Among children ages 2–14 years, the HIV positivity rate was 1.4%, adolescents ages 15–19 years had a positivity rate of 2.4% and adults ages 20 years and above had a positivity rate of 17.6%. Linkage rates of 92%, 73% and 72% for children, adolescents and adults, respectively, achieved with the HIV index testing model were higher than linkage rates observed with other HIV testing models. Results indicate that testing of biological children and sexual partners utilizing the HIV index testing model can be viable to identify and link children, adolescents and adults into care and treatment.

## 1. Introduction

Lesotho experiences an incredibly high HIV prevalence of 25.6% among adults ages 15–59 years, translating to 306,000 PLHIV in 2016 [1]. The HIV prevalence is at 2.1% amongst children ages 0–14 years representing 13,000 children living with HIV [1]. Despite an increase of antiretroviral therapy (ART) coverage among children from 43% in 2015 to 58% in 2016, there still remains a huge gap in diagnosis and initiation of ART among children in Lesotho [2]. Thus, there is an immediate need for scaled-up HIV Testing Services (HTS) to identify and link PLHIV with unknown status to care and treatment.

The HIV Index testing model involves provision of HTS to family members of known PLHIV (index clients) who are at increased risk of HIV infection such as sexual partners and children under 15 years. The model acknowledges the importance of providing HIV services to family members of an individual living with HIV for the following reasons: they are affected by HIV diagnosis of one family member; concerns around disclosure; stigma & discrimination; and treatment challenges [3, 4]. Not only does the index model demonstrate an increase in identification of HIV positive cases among children and adults, but it also leads to an increased linkage into care and treatment services [5, 6, 7].

One particular program in Abidjan, Cote d’Ivoire, worked with HIV-infected pregnant and postpartum women, as well as their families, to provide access to comprehensive HIV care and treatment. The program demonstrated successful enrolment of women living with HIV, their sexual partners and children in continuous follow-up care and treatment [8]. In Nyanza province of Kenya, the index model was particularly effective in testing children constituting 61% of family members that were identified and tested [5]. In addition, the index model contributes to identifying HIV at an earlier stage of infection, and improved health outcomes of all family members [9, 10, 11].

HIV Index testing further provides an opportunity for assisted disclosure in a family setting, closer rapport between HTS provider and HTS clients, and a higher HIV positivity rate [12]. However, disclosing the status of parents and testing children still remains a challenge. A study conducted in Uganda found dilemmas of parents disclosing their HIV status to their children due to fear of potential emotional trauma [13]. Many women fear of negative outcomes for disclosing their status including intimate partner violence [9, 14, 15]. Despite these barriers, disclosure can have positive health benefits as it has shown potential to increase social support and testing uptake while improving treatment adherence and retention [9, 14, 15]. The HIV index testing model has a huge potential to improve HIV status disclosure in families through HTS provider assistance.

Given its potential, it is crucial to overcome the aforementioned barriers and take advantage of the HIV index testing model. Community testing with targeted approaches to close the gap of testing opportunity missed by health facility based HTS is critical. The main objective of this study is to assess feasibility of the HIV index testing model in improving HIV positivity rate and linkage to care and treatment of sexual partners, adolescents and children of PLHIV in Lesotho.

## 2. Methods

### *2*.*1*. Introduction

Population Services International (PSI)/Lesotho with support from the Centers for Disease Control and Prevention (CDC) introduced the HIV index testing model in May 2015. PSI is a global health non-governmental organization (NGO) while the CDC is a United States governmental health protection agency. The model involves tracking and testing of biological children and sexual partners of known PLHIV. Index clients were identified from two sources; 1) selected high patient volume health facilities and; 2) all static, door-to-door and mobile PSI community-based HIV Testing sites (New Start Centers).

### *2*.*2*. Implementation sites

The HIV index testing model was implemented in five scale-up districts bearing 70% of the HIV burden in Lesotho (Maseru, Berea, Leribe, Mafeteng and Mohale’s Hoek). Index clients were identified from 18 high patient volume public health facilities, purposively selected with Ministry of Health (MOH) District Health Management Teams (DHMTs) involvement. These do not include all health facilities in the 5 scale-up districts. All PSI static, door-to-door and mobile HTS sites were also used as source of index recruitment process. Static sites allow walk-in clients to voluntarily come for HTS at fixed New Start Centers. PSI runs five fixed New Start Centers located in urban areas of the five scale-up districts. On the other hand, door-to-door testing masks two to three households adjacent to the identified index clients’ households. This method averts disclosure the index client's status by association; it is particularly used when branded vehicles associated with HTS are involved. In addition, mobile HTS outreach entails the provision of HTS in tents manned by HTS counsellors in the community.

### *2*.*3*. HIV index testing process

**Fig 1:**
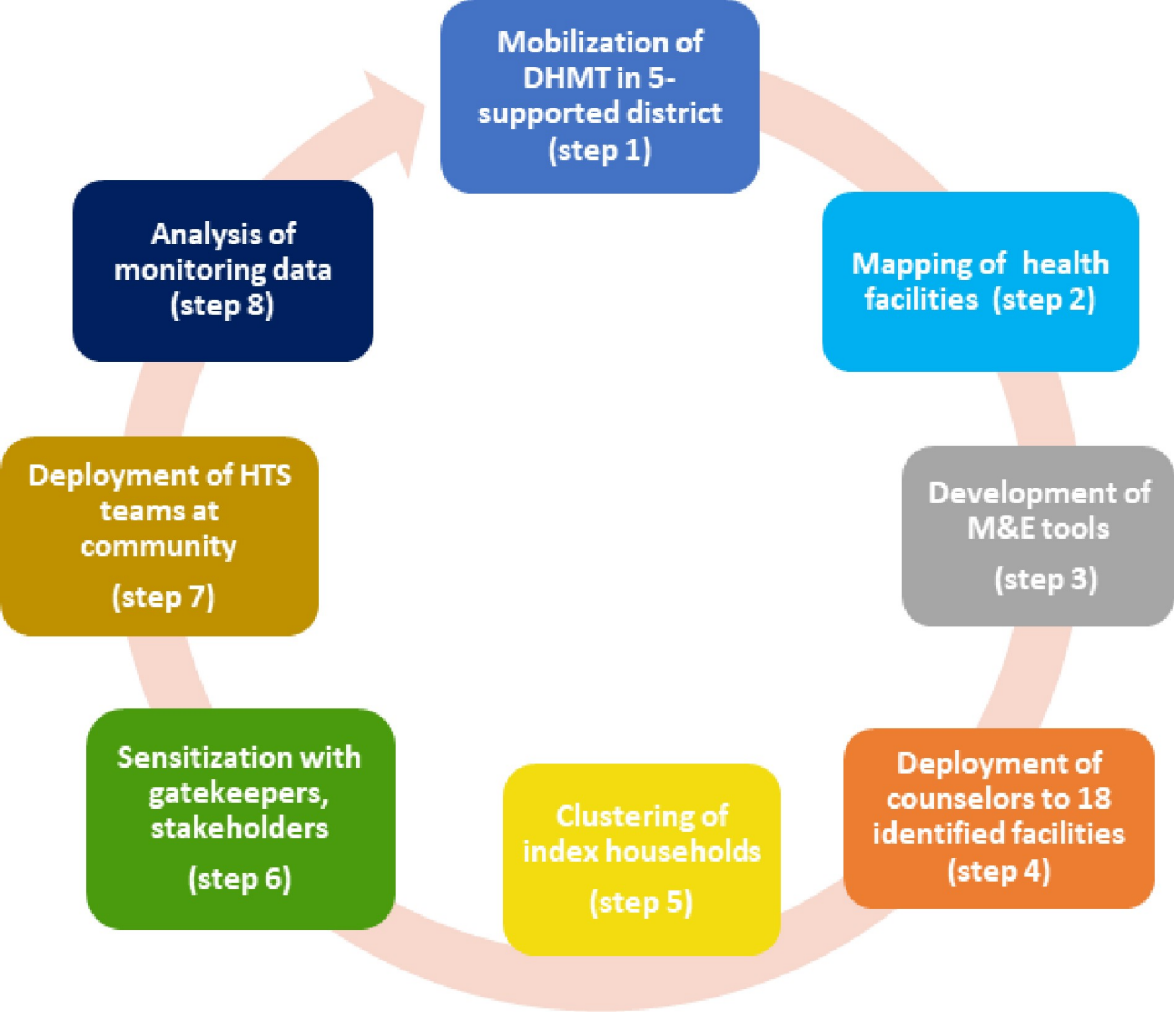
Steps for implementation of index model.

The MOH DHMTs were engaged regarding the need for HIV index testing in order to increase the HIV positivity rate of children and sexual partners of index contacts prior to implementation (see Fig 1). DHMTs assisted in identification of high patient volume facilities to implement HIV index testing based on HIV program data. Community gate-keepers including chiefs and constituency councilors were sensitized about HIV index testing in their communities.

PLHIV identified at facilities and through New Start HTS sites were offered home visits for the purpose of testing their biological children and sexual partners with unknown HIV status. The biological children of males were included for testing, especially when the mother’s status was unknown. All clients who consented to home visits were enrolled for HIV index testing. Once clients consented for the HIV index testing, an appointment was set for when the visit will be conducted. This was accomplished by HTS counsellors placed at health facilities and at New Start Centers.

HTS counsellors visited households identified as described above on the agreed appointment dates. HTS was provided to eligible clients in their homes. Rapid HIV Diagnostic Tests (Determine HIV 1/2 and Unigold) were used for HIV testing. The HTS counsellors provided masking door-to-door testing by providing HTS to three households in close proximity to the index client household. This approach helped to prevent ‘disclosure of HIV status by association’ with visit by HTS counsellors to a particular household.

Written informed consent was obtained from all HIV testing eligible clients. Written informed consent to test all children under age of 12 years were obtained from parents or guardians.

### *2*.*4*. Data collection

Bio-data forms that were developed collaboratively with the MOH HTS department were used to capture client data. The bio-data form collected information relating to the index client and the number of people who were likely to be tested during family testing. Relationship of the index client to the household member was established at the point of enrolment. This helped to identify biological children and sexual partners at increased risk of HIV exposure like children of the index clients and sexual partner(s). The HTS counsellors used this information to ensure that the members at increased risk received HTS.

A client intake record form was a tool used to collect information about clients during the testing process, such as demographic data, HIV testing history, HIV testing results obtained and preferred facilities for linkage to ongoing HIV care and treatment should the client be found to be living with HIV. This form was administered to all clients who were tested regardless of their positive or negative result. However, information of people who did not test was not collected. The known HIV positive children or sexual partners of an index client were not included in testing as we only tested people who either previously tested negative or did not know their status. All identified HIV positive clients received a referral form specifying the preferred health facility for ongoing care and treatment. Clients were encouraged to report to their preferred health care facility within one week of HIV diagnosis. The referral forms were filled in triplicate (one copy given to client to take to health facility, the second copy sent to referral and linkage coordinator and the third copy remained with the HTS counsellor). The data collection procedure was the same for other HIV testing models. Client intake forms were filled out for client data collection. The only difference is that all index contacts had a bio-data sheet that indicated the relationship to index client, where the index client was found, and whether the index client was already enrolled on ART.

Linkage is defined as the confirmation of newly diagnosed clients at the referral health facilities in regard to care and treatment services within three months of diagnosis [16]. Linkage was measured in a consistent manner for all HIV testing models. Referral & Linkage coordinators used the copies of completed referral forms received from HTS counsellors to track and establish client linkage into care and treatment. The referral and linkage coordinators visited health facilities stated on each referral forms for each identified HIV positive client to ascertain linkage. Linkage into care was considered if only the name of the client on the referral form was found in the facility held pre-ART or ART registers. The pre-ART number or Unique ART numbers were documented on the referral form copies to confirm linkage into care and treatment. Referrals for all clients that linked into care bearing either the pre-ART or Unique ART numbers were sent to Monitoring and Evaluation officers for data capturing.

Routine monitoring of data gathered between May 2015 and November 2017 was analyzed to assess feasibility and key features of this approach. All client data was kept in strict confidentiality and was stored in a password protected database. The HIV positivity and linkage rates from the index model were compared to other testing models implemented by PSI, including static site, door-to-door masking and mobile outreach HTS.

### *2*.*5*. Data analysis

HIV positivity rate is defined as percentage of the newly identified positives among all clients tested for HIV in the specific time. The numerator only includes newly identified positives during testing while the denominator includes all clients tested and excludes the known positives and those who declined testing.

Data was analyzed using the IBM Statistical Package Software for Scientists (SPSS), version20. Descriptive analysis using chi-square statistics was conducted with 5% level of significance.

### *2*.*6*. Ethical review

This analysis was conducted with routine data gathered through PSI/Lesotho HTS project. Informed consent for home visit was obtained for all PLHIV who served as index clients prior to home visit. Further informed consent was obtained for all clients who were tested for HIV through the HIV index testing model. Consent to test children below 12 years was obtained from parents or guardians in line with Lesotho HTS policy. Approval to use programmatic data was received from PSI. None of the authors had access to the data with identifiers. This study only analyzed anonymized and de-identified data.

## 3. Results

**Fig 2:**
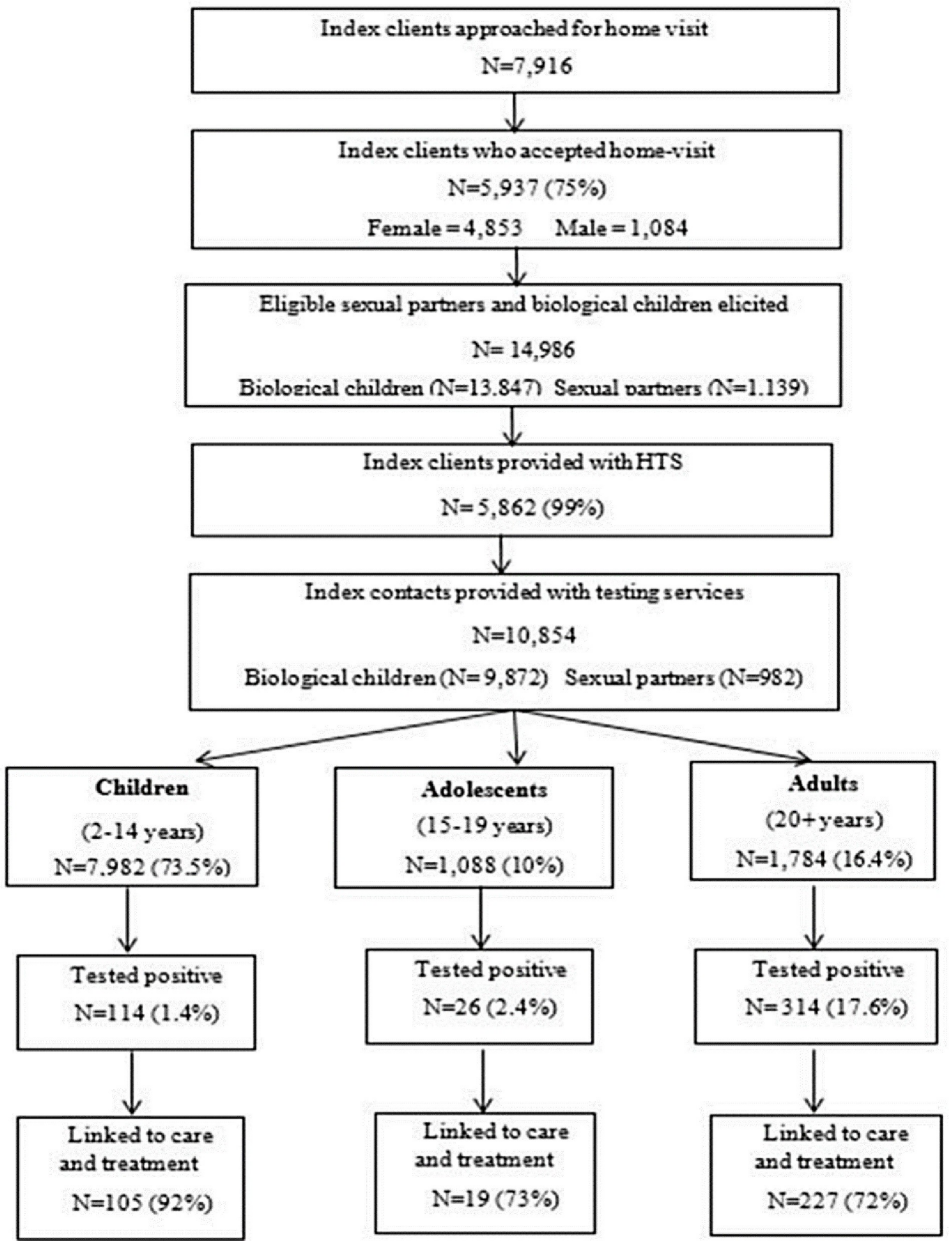
HIV testing services uptake in HIV index testing model from PSI/Lesotho.

A total of 7,916 clients diagnosed with HIV (index clients) were approached for family member testing (biological children and sexual partners) in five scale-up districts and 5,937 (75%) consented for home visits (Fig 2). Among all index clients who consented for home visits, 5,862 (99%) were provided with home testing. A total of 10,854 individuals were tested through HIV index testing. Biological children accounted for 91% (9,872) while sexual partners represented only 9% (982). Of all eligible family members elicited for testing, 72% (10,854/14,986) were tested for HIV. The proportion of eligible sexual partners getting tested at 86% (982/1,139) was much higher than that of eligible children getting tested at 71% (9,872/13,847).

Among the index contacts tested, 73.5% (7,982) were children ages 2–14 years and 10% (1,088) were adolescents ages 15–19 years. Adults aged 20 years and above accounted for 16.4% (1,784). Slightly above half (53.1%, 4,814) of all children and adolescent clients tested were female compared to 46.9% (4,256) male. Among adults 51.5% (918) tested clients were females and 48.5% (866) were males.

The overall HIV positivity rate among all index contacts (biological children, adolescents and sexual partners) who tested for HIV was 4.2% (454/10,854). Of the 454 PLHIV identified through HIV index testing, 25.1% (114/454) were children ages 2–14 years. Adolescents and adults represented 5.7% (26/454) and 69.2% (314/454), respectively. The HIV positivity rate by age group was 1.4% (114/7,982) among children ages 2–14 years, 2.4% (26/1,088) among adolescents ages 15–19 years and 17.6% (314/1,784) among adults (Fig 2).

HIV positivity rate was highest among sexual partners. Sexual partners ages 15–19 years had an HIV positivity rate of 22.6% compared to 1.8% among biological children, p = 0.001 (Table 1). The same pattern was observed with adults aged 20 years and above. Sexual partners aged 20 years and above had the highest HIV positivity rate at 24.9% compared to 9.2% of biological children in the same age group (p = 0.001). There were no children aged 2–14 years who were a sexual partner of index client.

**Table 1:** HIV positivity rate by age and relationship to index client: May 2015 to November 2017.

Age	Biological child	Sexual partner	Chi-Square	Significance level
2–14 years	1.4% (114/7,982)	Na	No measure of association	Na
15–19 years	1.8% (19/1,057)	22.6% (7/31)	55.77	p = 0.001
20+ years	9.2% (77/833)	24.9% (237/951)	75.25	p = 0.001

na = no data available

Children who received HTS for the first time had an HIV positivity rate of 1.9% compared to a 1.0% HIV positivity rate among children who ever tested for HIV, p = 0.001, as shown in Table 2. There was no difference in the HIV positivity rate among adolescents ages 15–19 years in relation to testing history. On the other hand, the HIV positivity rate was as high as 29.9% among adults aged 20 years and older who were first time testers compared to 14.8% among the same individuals who were re-testers, p = 0. 001.

**Table 2:** HIV positivity rate among children, adolescents and adults tested through HIV index testing model by testing history: May 2015 to November 2017.

Age	First-time tester	Re-tester	Chi-Square	Significance level
2–14 years	1.9% (73/3,931)	1% (41/4,051)	10.12	p = 0.001
15–19 years	1.7% (7/406)	2.8% (19/682)	1.23	p = 0.184
20+ years	29.9% (100/335)	14.8% (214/1,449)	42.68	p = 0.001

**Fig 3:**
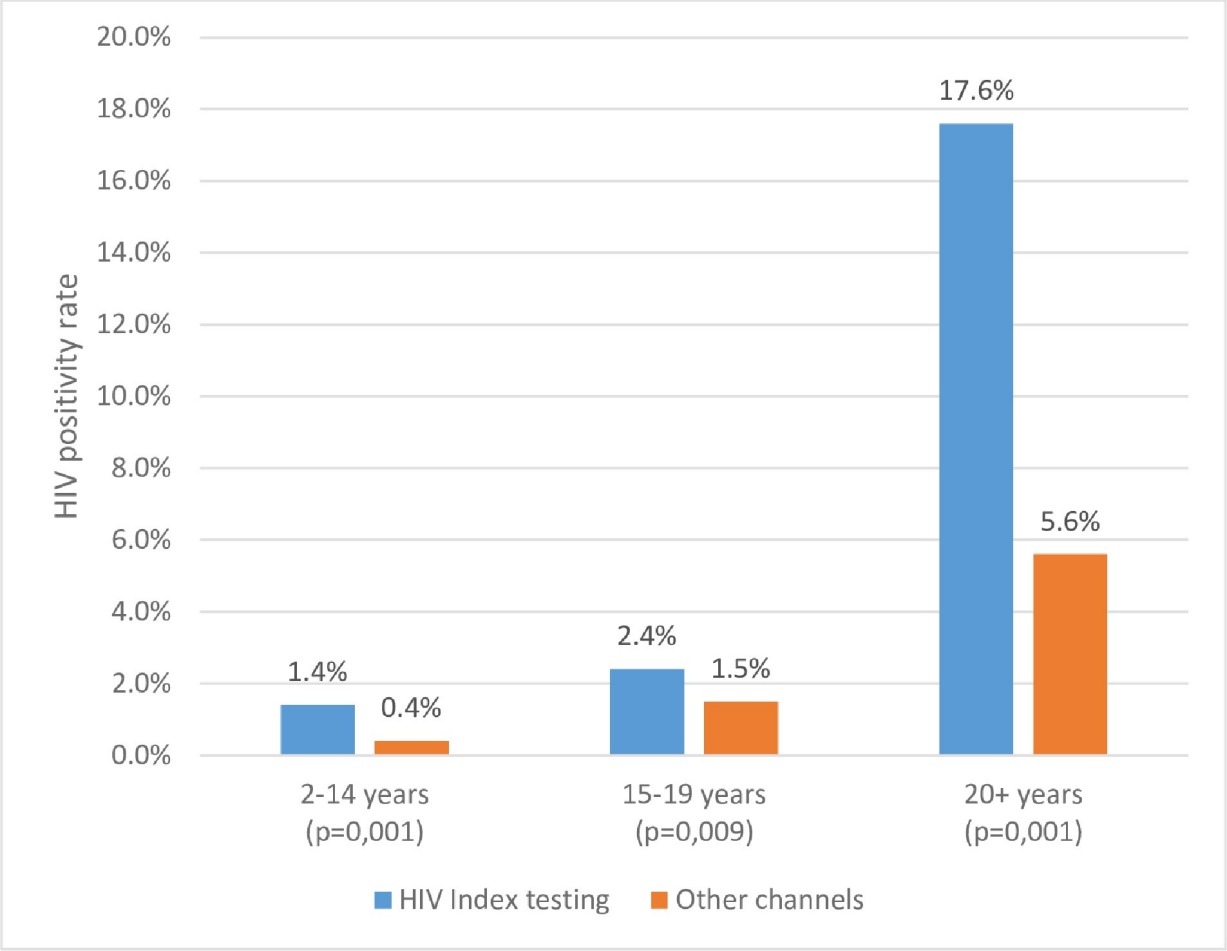
HIV positivity rate by age and HTS modality from May 2015 to November 2017.

The HIV positivity rate among children was higher with an HIV index testing rate of 1.4%, compared to 0.4% observed with other HTS models; p = 0.001 (Fig 3). A similar pattern was observed among adolescents with the HIV index testing model having higher HIV positivity rate of 2.4% compared to 1.5% at other HTS models; p = 0.009. Among adults, HIV index testing recorded a higher HIV positivity rate, 17.6%, compared to the 5.6% HIV positivity rate obtained during other HTS models; p = 0.001.

The HIV positivity rate was higher among children identified through newly diagnosed index clients compared to those identified through index clients already on ART (4.0% vs. 1.3%; p = 0.001) as shown in Table 3 below. However, there was no statistical difference observed in the HIV positivity rate between adolescents tested through newly diagnosed index clients and those already enrolled on ART. Among adults aged 20 years and above, the positivity rate was also higher among clients identified through newly diagnosed PLHIV index clients at 31% compared to a 16.1% HIV positivity rate observed among clients identified through index clients already on ART (p = 0.001). For all three age groups, the HIV positivity rate was higher among clients identified through newly diagnosed index clients compared to clients identified through index clients already on ART (11.3% vs. 3.7%; p = 0.001).

**Table 3:** HIV positivity rate among children, adolescents and adults by index client type (Newly diagnosed Index Clients vs. Index Clients on ART): May 2015 to November 2017.

Age	Contacts of newly identified Index client	Contact of already enrolled index client	Chi-Square	Significance level
2–14 years	4.0% (17/427)	1.3% (97/7,555)	20.89	p = 0.001
15–19 years	4.4% (2/45)	2.3% (24/1,043)	0.85	p = 0.293
20+ years	31.0% (54/174)	16.1% (260/1,610)	23.99	p = 0.001

**Fig 4:**
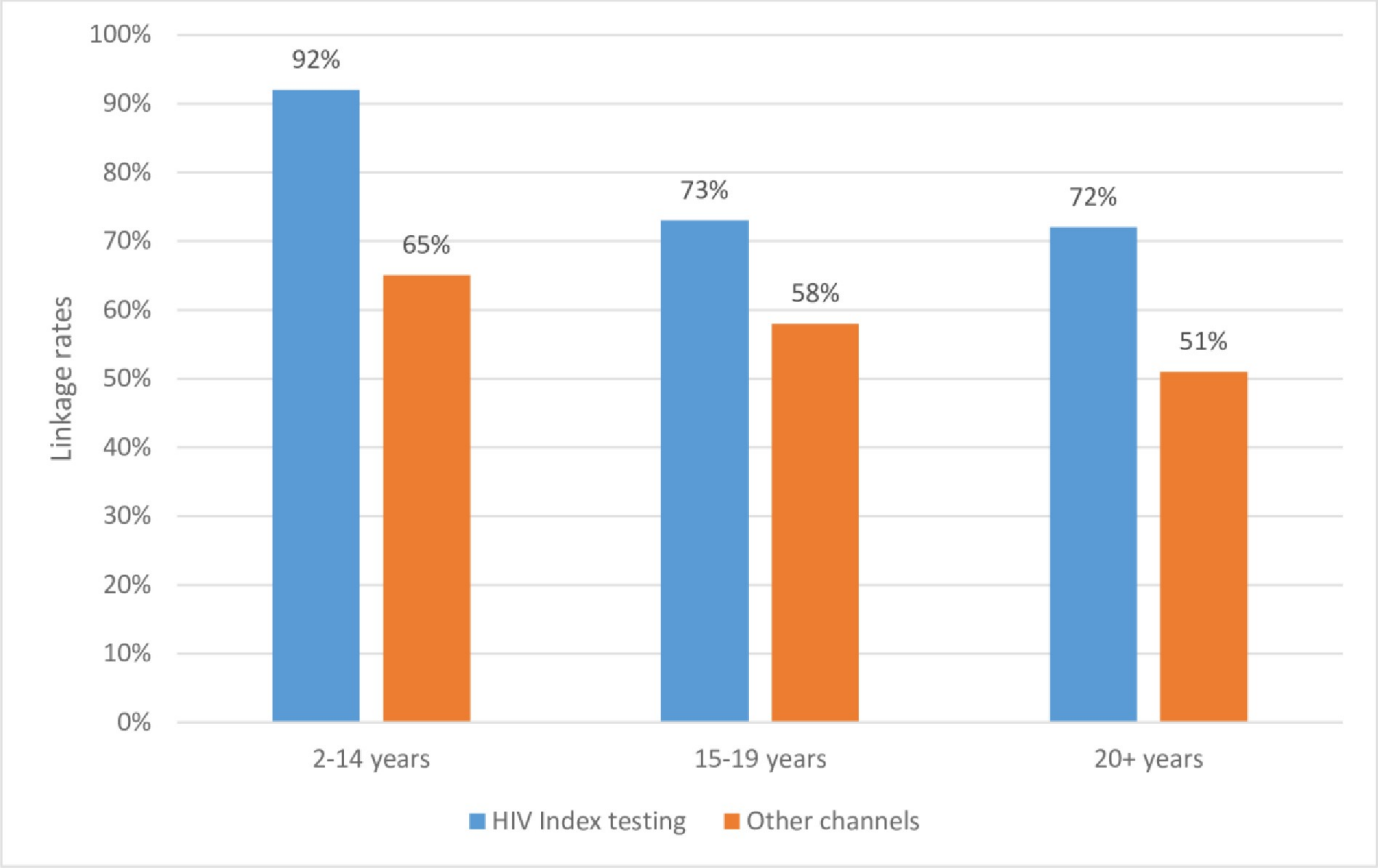
Linkage rates by HTS modality and age from May 2015 to November 2017.

Successful linkage to care and treatment for children living with HIV was higher with HIV index testing at 92% compared to 65% among children tested with other HTS models (Fig 4). Analysis of linkage rates for adolescents living with HIV showed highest linkage among those identified through HIV index testing at 73% compared to 58% for other HTS models. A similar pattern was observed among adults newly identified as HIV positive with a linkage rate of 72% among those tested through HIV index testing, compared to 51% linkage rate for other HTS models.

## 4. Discussion

We found that HIV index testing was effective in the provision of HTS to biological children and sexual partners of PLHIV who received home visits through the outreach approach. This finding was consistent with observations made in Kenya which found that an index model increased HIV testing among family members [5]. Results in this study indicated that HIV positivity rate among sexual partners was the highest. This aligns with a study conducted in Tanzania using partner notification approach [17]. It was further observed in this study that the HIV positivity rate was higher among children and sexual partners of index clients who were first time testers than those who had negative HIV status before. This may be because first time testers perceive themselves to be at lower risk of infection [18]. Further, re-testers may not expose themselves to risk behaviors after knowing their negative status. In this study, for HIV positive results, we only included the newly diagnosed HIV positive results and those who reported previous HIV positive were excluded.

Results also indicate that the HIV index testing model can be a viable method for reaching HIV-positive sexual partners and children who have not yet been diagnosed. Adults and children identified through newly diagnosed index clients had a higher positivity rate than those who were diagnosed through index clients already enrolled on ART. This may reflect the preventive effect of use of ART for Prevention of mother-to-child transmission (PMTCT) and other prevention efforts [19]. Index clients who were already on ART might be virally suppressed, leading to lower transmission rates of HIV to their biological children and sexual partners.

The study also shows high linkage rates with the HIV index testing model than other HTS channels implemented at community settings. There is a remarkably high linkage rate among children, which confirms findings from earlier studies. Studies done in Kenya and South Africa demonstrated that the family-centered model leads to more success in finding HIV positive children and linking them to care and treatment [5, 6, 7]. Research conducted in South Africa showed that family centered HIV services including HTS results in better care and treatment outcomes for children living with HIV [20]. The high enrolment in care and treatment services maybe consequent to a more personal engagement of parents and guardians of children living with HIV by HTS providers at household level. Sexual partners identified as HIV positive through HIV index testing had high linkage rates. This agrees with study conducted in Tanzania using the partner notification approach [17]. The index HIV Testing model demonstrates high potential to improve ART coverage in Lesotho among children and adults through timely diagnosis and linkage to care and treatment.

### 4.1. Strengths and limitations

A major strength of this analysis was the large sample size of 7,982 children ages 2–14 years, 1,088 adolescents ages 15–19 years and 1,784 sexual partners included in the analysis. All clients at high volume health facilities offering HTS and HIV care and treatment services were included in the study.

However, some challenges were experienced during field visits where other family members to be tested were not present at the time of home visit by HTS counsellors. Another challenge was that parents were sometimes not available to give consent for testing for their children below age of consent, 12 years. In order to remedy this challenge, index clients were mostly appointed during weekends when both parents and children would most likely be available. Service hours were adjusted to accommodate clients’ schedules by offering HIV Index testing during weekends, public holidays and after-hours.

Another limitation of this study is that it was implemented only in five high HIV prevalence scale-up districts with more resources allocated. The other five maintenance districts were not included. Self-reported history of previous HIV testing results could potentially add recall bias.

Similar studies are required in maintenance districts of Lesotho to ascertain inter-district variability of HIV Index Testing outcomes. The study points towards the need to establish any other routes of HIV transmission including sexual assault particularly for newly diagnosed children below 15 years of age who had previous HIV negative results. Future research should examine the higher HIV positivity rate among first time testers compared to re-testers in context of Lesotho.

### 4.2. Recommendations

In order to maximize this approach, counsellors need to follow up with all newly identified HIV positives as early as possible. Complete recording of client biodata is critical for successful implementation of HIV Index Client Testing model. Post-test counselling for newly identified PLHIV should include the importance of provision of HIV testing to at high-risk family members (biological children and sexual partners) to avoid missing clients living with HIV. The use of re-testing screening should be emphasized to HTS providers so that testing is not routine for those not at high risk of acquiring HIV especially children who are not yet sexually active.

Health care providers should routinely offer HIV index testing as part of HIV care service package. Utilizing HIV index testing for biological children and sexual partners could be effective in identifying HIV positive clients at an early stage of infection thus contributing to positive health outcomes [9, 10, 11].

## 5. Conclusions

The HIV Index testing model produced higher HIV positivity and linkage rates across all age groups compared to other HTS models in community settings. This makes the model a viable approach to enhance identification of PLHIV and linking them to care and treatment.

## Supporting information

S1 Dataset (XLSX)
